# Dynamics of an Interactive Network Composed of a Bacterial Two-Component System, a Transporter and K^+^ as Mediator

**DOI:** 10.1371/journal.pone.0089671

**Published:** 2014-02-28

**Authors:** Ralf Heermann, Katja Zigann, Stefan Gayer, Maria Rodriguez-Fernandez, Julio R. Banga, Andreas Kremling, Kirsten Jung

**Affiliations:** 1 Center for Integrated Protein Science Munich (CiPSM) at the Department Biology I, Microbiology, Ludwig-Maximilians-Universität München, Martinsried, Germany; 2 Fachgebiet für Systembiotechnologie, Technische Universität München, Garching b. München, Germany; 3 BioProcess Engineering Group, IIM-CSIC, Spanish Council for Scientific Research, Vigo, Spain; University of Cambridge, United Kingdom

## Abstract

KdpD and KdpE form a histidine kinase/response regulator system that senses K^+^ limitation and induces the *kdpFABC* operon, which encodes a high-affinity K^+^ uptake complex. To define the primary stimulus perceived by KdpD we focused in this study on the dynamics of the Kdp response. *Escherichia coli* cells were subjected to severe K^+^ limitation, and all relevant parameters of the Kdp response, i.e., levels of *kdpFABC* transcripts and KdpFABC proteins, as well as extra- and intracellular K^+^ concentrations, were quantitatively analysed over time (0 to 180 min). Unexpectedly, induction of *kdpFABC* was found to follow a non-monotonic time-course. To interpret this unusual behaviour, a mathematical model that adequately captures the dynamics of the Kdp system was established and used for simulations. We found a strong correlation between KdpD/KdpE activation and the intracellular K^+^ concentration, which is influenced by the uptake of K^+^ via the KdpFABC complex. Based on these results a model is proposed in which KdpD/KdpE phosphorylation is inversely correlated with the intracellular K^+^ concentration. To corroborate this hypothesis an isogenic mutant that produces a defective KdpFABC complex, and the *trans*-complemented mutant that expresses the KtrAB high-affinity K^+^ uptake system of *Vibrio alginolyticus* were quantitatively analysed. Experimental data and simulations for the mutants consistently support the tight correlation between KdpD/KdpE activation and the intracellular K^+^ concentration. This study presents a striking example of the non-intuitive dynamics of a functional unit comprising signalling proteins and a transporter with K^+^ as mediator.

## Introduction

An adequate supply of the monovalent cation K^+^ is vital for the survival of all living organisms. It is required for the regulation of cell turgor [1] and pH homoeostasis [2], for activation of various enzymes [3] or transporters [4], for gene expression [5]–[7], translation [8], and the regulation of stress responses [9], [10]. *E. coli* possesses three K^+^ uptake complexes. Two of these, TrkG/TrkH and Kup, are constitutively produced and have low affinities for K^+^ [11], [12]. The third one, the high-affinity K^+^ transport complex KdpFABC, is only induced when the other transporters are unable to supply the cell's requirement for K^+^ [1], [13], [14]. This situation occurs under conditions of K^+^ limitation (at extracellular K^+^ concentrations <2 mM), mutation of *trk*, or under hyperosmotic stress imposed by NaCl [15].

**Figure 1:**
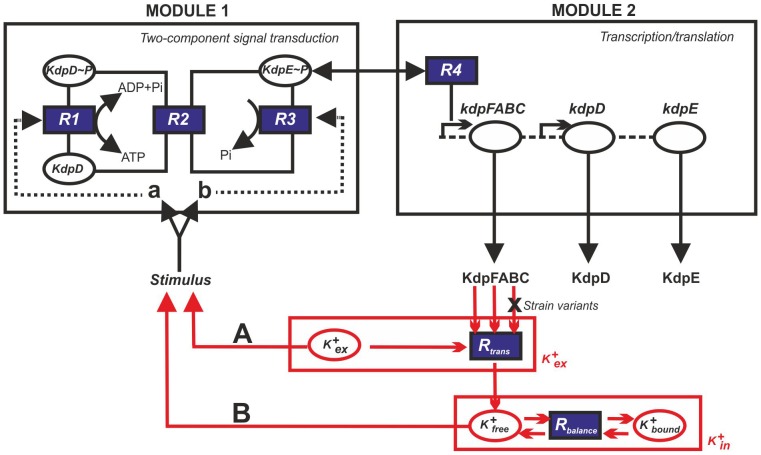
Basic scheme used to construct the mathematical model of the Kdp system. The model is made up of two modules: two-component signal transduction and transcription/translation. The input to the first module is the activating stimulus for KdpD, which results in phosphorylated KdpE, the input to the second module. The output of this module is the production of KdpFABC. The two modules are also linked by a feedback loop from KdpFABC to the KdpD/KdpE system with K^+^ as the mediator. This model reflects the uptake of external K^+^ (K^+^_ex_) by KdpFABC and the concomitant effects on the balance of intracellular K^+^ in the bound (K^+^_bound_) and free form (K^+^_free_). Two potential primary stimuli for KdpD are considered: the external K^+^ concentration (**A**) and the internal K^+^ concentration (**B**). Furthermore, K^+^ may affect either the kinase (**a**) or the phosphatase activity (**b**) of KdpD.

Expression of the *kdpFABC* operon is controlled by the histidine kinase/response regulator system KdpD/KdpE [16], [17] (Fig. 1). Among two-component systems, the KdpD/KdpE pair is the most widespread. Homologous systems have been found in more than 1000 bacterial and archaeal species, including many pathogens [18]. Upon activation, the histidine kinase KdpD autophosphorylates and transfers the phosphoryl group to the response regulator KdpE by means of its intrinsic kinase activity [19]. Phosphorylated KdpE exhibits increased affinity for the *kdpFABC* promoter and thereby triggers transcription of the operon (Fig. 1) [20]. The enzymatic activities of purified KdpD and KdpE have been characterized *in vitro* [21]. In particular, KdpD has been shown to be the only protein that dephosphorylates phospho-KdpE and, consequently, it also turns off *kdpFABC* expression (phosphatase activity) (Fig. 1) [21].

The nature of the primary stimulus perceived by KdpD remains contentious. Epstein and coworkers proposed that KdpD senses a decrease in turgor or some effect thereof [15], [22]. However, measurements of the volume of cells exposed to different external osmolytes have revealed that reduction of turgor cannot be the primary stimulus for KdpD [23]. Various groups have argued for [K^+^] as the control signal for KdpD, and suggested that the primary stimulus might be either the level of intracellular K^+^, processes associated with K^+^ transport, or the external K^+^ concentration [24]–[26]. It is important to note that the level of *kdpFABC* expression is at least ten-fold higher under K^+^ limitation than that induced in response to salt stress, which argues for a specific effect of K^+^ on KdpD [23], [27]. The observation that extracellular Cs^+^, which is taken up and significantly reduces the availability of free intracellular K^+^, induces *kdpFABC* expression [27] also supports this idea. Furthermore, it has been reported that the fall in internal K^+^ concentrations seen in *E. coli* exposed to acid stress is accompanied by an increase in *kdpFABC* expression [28]. *In vitro* phosphorylation assays based on right-side-out membrane vesicles have revealed an inhibitory effect of K^+^ on the kinase activity of KdpD [29]. An inhibitory effect of K^+^ has also been observed during the *in vitro* reconstitution of the complete signal transduction cascade, consisting of KdpD in proteoliposomes, purified KdpE, a DNA fragment comprising the KdpE-binding site, and a mixture of ATP/ADP. The higher the K^+^ concentration, the lower the level of phosphorylated KdpE [30], [31]. These data argue for an effect of the intracellular K^+^ concentration on the activity of KdpD. However, more recent studies have indicated that KdpD activation cannot be solely attributed to the intracellular K^+^ content, and that the extracellular K^+^ concentration also plays a role [32].

To complete this picture it should be noted here that KdpD activity is also modulated by alterations in the ionic strength [29] and by ATP via a regulatory ATP-binding site [33], [34]. Furthermore, under conditions of salt stress, UspC provides a scaffold for the KdpD/KdpE signalling cascade and thereby supports phosphorylation in this particular context [30], [35].

Thus the data collected over the years have not provided a definitive answer to the question of the nature of the primary stimulus for the KdpD/KdpE system. This is largely because it is difficult to determine the various functional states of KdpD and KdpE *in vivo*. Quantitative analyses of components of biochemical reaction pathways are generally problematic, and represent a real challenge in the life sciences. Mathematical modelling, which allows one to reconstruct biochemical reaction networks *in silico*, provides one way around this impasse. Especially in cases where there is uncertainty about interactions between biochemical compounds, model analysis can help to decide whether certain network topologies can explain experimental observations. Given some prior knowledge or assumptions about the reactions they are involved in, the dynamic behaviour of unmeasured parameters can be simulated. Hence such models can also yield virtual “measurements” of these networks.

To elucidate how K^+^ affects the KdpD/KdpE system *in vivo*, we combined a quantitative experimental analysis with mathematical modelling. First we extended and modified an existing model of the Kdp system (Fig. 1) [36]. Then, we directly monitored and theoretically modelled the time-resolved dynamics of the most salient parameters of the Kdp system in the wild type strain and in selected mutant strains. Our data provide, for the first time, a detailed picture of this highly dynamic interactive network involving two signalling proteins and one K^+^ transporter, in which K^+^ ions serve as major control parameter during adaptation of *E. coli* to persistent stress under K^+^ limitation.

## Results

### Description of the Kdp system

The Kdp system was previously described by a model consisting of two modules linked by a black box representing a negative feedback loop from KdpFABC to KdpD/KdpE (Fig. 1) [36]. Specifically, the first module describes the autophosphorylation of KdpD, transfer of the phosphoryl group from phospho-KdpD (KdpD-P) to KdpE, dephosphorylation of phospho-KdpE (KdpE-P) yielding inorganic phosphate, and binding of KdpE-P to the *kdp* promoter. The second module accounts for the processes leading to KdpFABC production, and describes the changing numbers of *kdpFABC* transcripts per cell, based on rates of synthesis and degradation of the *kdpFABC* mRNA, and rates of synthesis, turnover and destruction of the proteins KdpFABC, KdpD and KdpE. The feedback loop linking KdpFABC to the first module is now elaborated by a more detailed dynamic model that incorporates the uptake of external K^+^ by KdpFABC and changes in the sizes of free and bound intracellular K^+^ pools (Fig. 1).

### Time-resolved dynamics of the Kdp system in *E. coli*

**Figure 2:**
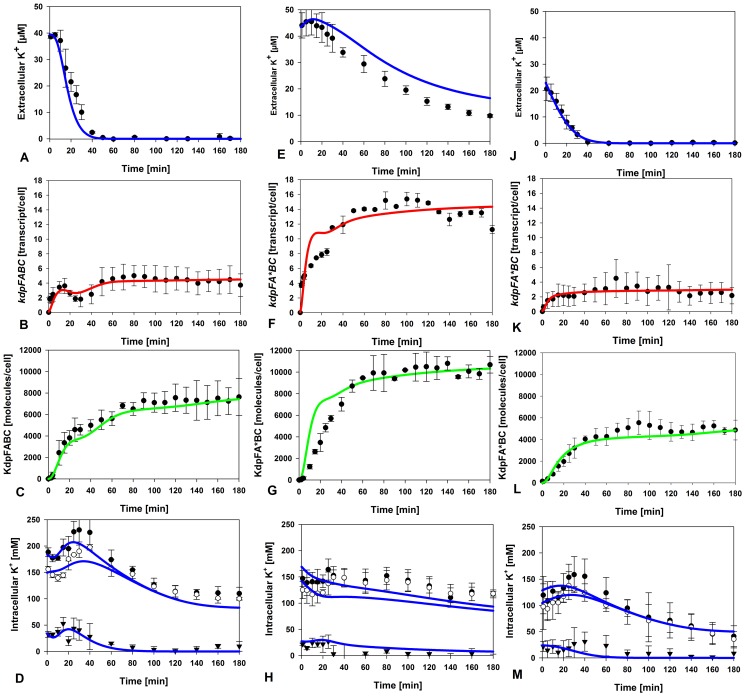
Induction kinetics of the Kdp system in *E. coli*. Exponentially growing cells were shifted to limiting K^+^ (40 µM K^+^) at time 0. At the indicated times thereafter cells were harvested, and extracellular and intracellular K^+^ concentrations were determined by atomic absorption spectroscopy, *kdpFABC* expression was measured by qRT-PCR, and KdpFABC production by quantitative Western blot analysis. Three different strains were used: MG1655 (**A–D**), RH010, which expresses KdpFA(G345S)BC and is defective in K^+^ transport (**E–H**), and RH010 transformed with plasmid pKT84, which encodes the high-affinity K^+^ transport system KtrAB (**J–M**). (**A, E, J**) Extracellular K^+^ concentration; (**B, F, K**) *kdpFABC* transcripts; (**C,G,L**) KdpFABC proteins; (**D, H, M**). Intracellular K^+^ concentrations: K^+^_total_ (•), K^+^_bound_ (○), K^+^_bound_, and K^+^_free_ (▾). Results of simulations based on the model are presented by the coloured lines: K^+^ concentrations (blue), transcripts (red), KdpFABC proteins (green). All experimental data are average values of at least three independent experiments, and error bars represent the standard deviation of the mean.

To obtain a time-resolved dataset, samples were collected from an exponential-phase culture of *E. coli* MG1655 growing in a medium containing 10 mM K^+^ and transferred to conditions of severe K^+^ limitation (0.04 mM K^+^) at time zero (Fig. 2). The extracellular K^+^ concentration remained nearly constant for the first 10 min after the shift (Fig. 2A). Then, concomitantly with rising numbers of KdpFABC complexes, K^+^ vanished from the medium at a constant rate, and was completely used up after 45 min (Fig. 2A). This behaviour can be attributed to transport of the ion by the high-affinity K^+^ uptake system KdpFABC, since the constitutively produced Trk (K_m_ between 1 to 2 mM [11]) is essentially unable to transport K^+^ into the cell under these conditions, while the third K^+^ import system Kup has no impact on rates of K^+^ uptake at neutral pH [37]. These constraints may also account for the initial delay in the onset of uptake after transfer.

The changes in the number of *kdpFABC* transcripts per cell over time were quantified by qRT-PCR (Fig. 2B). Transcription was induced immediately after the shift, and transcript levels increased to reach a maximum after 15 min. Thereafter, transcript levels fell by about 50% to reach a minimum at t = 30 min. At t = 40 min, *kdpFABC* transcript numbers began to rise again, ultimately attaining a maximum that was approximately 40% higher than the first peak. This level was then maintained until the end of the experiment at t = 180 min (Fig. 2B).

In parallel, we used quantitative Western blot analysis to measure the numbers of KdpFABC molecules translated from *kdpFABC* transcripts (Fig. 2C). After a short lag period (the time required for protein synthesis), the number of KdpFABC complexes steadily increased in cells exposed to K^+^ limitation. Within 30 min a level of about 5,000 molecules per cell was reached. Then the net rate of production of KdpFABC complexes decreased, coincidentally with the drop in the numbers of *kdpFABC* transcripts per cell, and was maintained at that level until t = 90 min. The number of KdpFABC complexes showed no further increase thereafter, remaining at a level of about 8,000 per cell. Thus, the net rate of synthesis of KdpFABC complexes followed a hyperbolic time-course.

To complete the dataset, we also measured the changes in intracellular K^+^ concentration. It is important to note that the level of free intracellular K^+^ fell markedly (from 320 mM to about 200 mM, see also Fig. 2D) during the time taken to shift the cells into the low-K^+^ medium. Within the first minutes the total intracellular K^+^ concentration continued to decline further, and then rose for a short period (t = 5–30 min), and declined again thereafter. It is generally accepted that a significant fraction of the intracellular K^+^, referred to as the “bound” fraction, is associated with anionic macromolecules. This fraction has a much reduced osmotic and ionic activity, and can therefore be distinguished experimentally from the “free” K^+^ fraction [38]. The fraction of free intracellular K^+^ increased after a lag time, and remained at a plateau level over the next 20 min. Thereafter, this fraction decreased at a constant rate, and was barely detectable at t = 70 min. These results reveal a clear inverse correlation between *kdpFABC* transcript number and the concentration of free K^+^ ions in the cell (see Fig. 2B and D), and it is hypothesized that free intracellular K^+^ ions have a negative feedback regulatory effect on the KdpD/KdpE system.

### Time-resolved dynamics of the Kdp system in a Kdp transport-defective mutant

To test this hypothesis further, we wished to analyse a mutant in which K^+^ uptake via the KdpFABC complex was compromised. For this purpose, we constructed *E. coli* strain RH010 (MG1655 *kdpA4*), which encodes a KdpFABC complex with an altered K^+^-binding site [39], [40]. This strain has a chromosomal point mutation in *kdpA* (Gly345 to Ser), which replaces a glycine in the K^+^ selectivity filter III by a serine residue. Replacement of this amino acid reduces the affinity for K^+^ by about 30-fold [41]. Therefore, this mutant has very little capacity to transport K^+^ under conditions of K^+^ limitation. The strain also carries a chromosomal point mutation in the *rpsL* gene, which confers streptomycin resistance and was used for selection during strain construction [40]. Earlier tests had indicated that this mutation has no effect either on *kdpFABC* transcription or KdpFABC synthesis (data not shown).

*E. coli* RH010 cells were subjected to the same severe K^+^ limitation as the wild type strain. In these cultures the extracellular K^+^ concentration also decreased over time, but at a much lower rate relative to the wild type (Fig. 2E), probably due to the low activity of the KdpFABC-G345S complex. It should be mentioned that *E. coli* RH010 grows slowly under these conditions. The peak concentration of free intracellular K^+^ reached in the mutant was only half that seen in the wild type (Fig. 2H). In contrast, the initial burst in the synthesis of *kdpFABC* transcripts attained a plateau that was about three-fold higher than in the wild type (Fig. 2F). Thus, unlike the case in wild type, the time-course of *kdpFABC* transcription in the mutant follows a hyperbolic curve.

Although the mutant produced three times as many transcripts, the maximal number of KdpFABC molecules reached in the RH010 strain was only 1.5-fold higher than in wild type (Fig. 2G). To clarify the reasons for this discrepancy, pulse-chase experiments using [^35^S]methionine were performed to compare the stability of the wild type and mutant KdpFABC complexes (Fig. S1). The half-lives of the KdpFABC complex and its KdpFABC-G345S derivative were found to be 36.7 min and 28.4 min, respectively, indicating that the mutant complex is indeed less stable than the wild type.

To probe the relationship between intracellular K^+^ and *kdpFABC* expression further, we introduced a second high-affinity K^+^ uptake system into *E. coli* RH010. The strain was transformed with plasmid pKT84 encoding the high-affinity KtrAB system from *Vibrio alginolyticus* [42] (the K_M_-value of KtrAB is in the µM-range as for KdpFABC), and the transformants were treated and analysed as before. Because expression of *ktrAB* is constitutive, *E. coli* RH010 cells producing KtrAB consumed the supply of extracellular K^+^ much faster than the wild type, which is indicated by a significant drop of the extracellular K^+^ concentration in the initial phase (Fig. 2J). For reasons that remain unclear, however, the total concentration of intracellular K^+^ immediately after the shift was lower than in the wild type (Fig. 2M). Importantly, the numbers of *kdpFABC* transcripts and KdpFABC complexes per cell at steady state were comparable to those seen in the wild type (Fig. 2K, 2L), again indicating a negative feedback effect of internal K^+^ on KdpD/KdpE activation. However, the dynamics of *kdpFABC* transcription of the *trans*-complemented RH010 mutant differed from the wild type (compare Fig. 2B and Fig. 2K). The discrepancy can be explained by the fact that expression of *ktrAB* is constitutive.

In summary, the time-resolved dynamics of *kdpFABC* transcripts and KdpFABC complexes differ significantly in *E. coli* wild type and mutant RH010. Overall, the data argue for negative feedback regulation by free intracellular K^+^ under severe K^+^ limitation, which modulates KdpD/KdpE phosphorylation and thus affects *kdpFABC* expression.

### Design of an extended mathematical model

The phosphorylation state of the response regulator KdpE is directly controlled by the enzymatic activities of the cognate sensor kinase KdpD [21]. Having demonstrated that the intracellular K^+^ concentration influences *kdpFABC* expression under severe K^+^ limitation, we were interested in determining the effect of K^+^ on KdpD/KdpE phosphorylation over the whole environmental induction range (0.04–2 mM K^+^). As the enzymatic reactions involved occur too rapidly for experimental investigation in vivo, we used mathematical modelling to address this issue. First we had to extend the existing model of the Kdp system [36], taking the experimental data obtained for the wild type and the RH010 mutant into account. Thus, we added mass balance equations to describe the temporal alterations in external [K^+^] and intracellular K^+^ concentrations (free and bound) as well as the total cell volume in our samples. The detailed derivation of the full model is set out in Materials and Methods. The relevant intracellular state variables in the model are concentrations, and thus depend on the volume of viable cells present at any given point. Potential alterations in cytoplasmic volumes were analysed over time, but the differences between stressed (40 µM K^+^) and non-stressed (10 mM K^+^) cells were found to be marginal (Table S1). An experimental comparison between total and viable cell numbers after exposure to K^+^ limitation revealed that approximately 60% of the cells survived the shift and continued to grow (Table S1). Similar values were obtained for mutant RH010 (data not shown).

The mathematical model was then used to study how K^+^ levels influence KdpD/KdpE activation and thus transcription of the *kdpFABC* operon. Since the level of phosphorylated KdpE and, consequently, the number of *kdpFABC* transcripts, seems to be a measure of the K^+^ concentration, it can be assumed that K^+^ (external or internal, Fig. 1, options **A** or **B**) either inhibits the kinase or activates phosphatase activity of KdpD (Fig. 1, options **a** or **b**). One can deduce several possible network topologies from these considerations: (i) external K^+^ (*K^+^_ex_*) inhibits KdpD autophosphorylation; (ii) free intracellular K^+^ (*K^+^_free_*) inhibits KdpD autophosphorylation; (iii) *K^+^_ex_* enhances dephosphorylation of KdpE-P, or (iv) *K^+^_free_* enhances dephosphorylation of KdpE-P. Moreover, these effects may act in combination: (i) *K^+^_ex_* inhibits the kinase activity and *K^+^_free_* enhances the phosphatase activity of KdpD, or (ii) vice versa. Therefore, we generated several model variants (with standard parameters from the original model and from the literature) that depicted these different network topologies and tried to calibrate them against the experimental data for the wild type and the RH010 mutant strain (Fig. 2A–H). We then used computer simulations to infer which of the conceivable networks most faithfully reproduced the experimental data. We found that the experimentally observed non-monotonic dynamic behaviour of *kdpFABC* transcription could best be explained by a positive feedback effect of the free intracellular K^+^ on the dephosphorylation of KdpE-P, i.e. K^+^ activates the phosphatase activity of KdpD. This variant yielded the best quantitative match between experimental and simulated data. Hence, in the following, the term “the model” always refers to this topology. All other topology variants tested were far less successful in reproducing the experimental data.

This finding is quite realistic for the following reasons. On the one hand, our simulations showed that KdpD/KdpE could not respond fast enough to the changes in both external and intracellular K^+^ pools if the control operated at the level of autophosphorylation alone. Furthermore, the maxima and minima of the experimentally determined time-courses of *K^+^_free_* and *kdpFABC* transcripts are inversely correlated. In contrast, external K^+^ is decreasing at a constant rate, so that this parameter is unlikely to drive non-monotonic transcript dynamics.

Finally, the model parameters had to be tuned to replicate the data reported above for wild type and the RH010 mutant strain (Fig. 2A–H), i.e. to adequately reproduce the temporal behaviour of the *kdpFABC* transcripts, the KdpFABC complexes and the different K^+^ pools (external, free and bound intracellular). Initial calibration was performed by manual adjustments made by the modeller during the simulations to improve the fit to the experimental data (see Materials and Methods). Fine-tuning of the parameters was then done using SensSB software [43], a Matlab (www.mathworks.com) toolbox that integrates different methods for sensitivity analysis and parameter estimation of dynamic models. All parameters used are summarized in Table 1.

**Table 1:** Optimized parameters of the mathematical Kdp model.

Parameter name	Value			Units	Description
	Wild type	*E. coli* RH010	*E. coli* RH010/pKT84		
*Two-component system*					
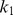	0.23			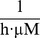	Autophosphorylation of *D*, forward reaction rate constant
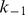	5.1×10^−6^			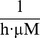	Autophosphorylation of *D*, backward reaction rate constant
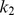	2.27×10^3^			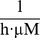	Phosphotransfer to *E*, forward reaction rate constant
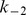	8.7×10^−4^			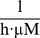	Phosphotransfer to *E*, backward reaction rate constant
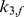	40.6×10^−3^			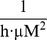	Dephosphorylation of *E^P^* by *D*
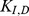	520			mM	Inhibition of autophosphorylation of *D* by free K^+^
*Transcription*					
*K*	4×10^4^	1.2×10^4^	6×10^4^	1	Equilibrium binding constant of σ-factor and RNA polymerase to DNA
*K_E_*	5.32×10^−2^			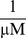	DNA-binding of free *E^P^*, equilibrium dissociation constant
*α*	2.59×10^−3^			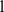	Affinity factor
*k_tr_*	1.06×10^4^			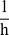	Transcription rate constant
*k_z_*	21.74			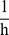	Transcript degradation rate constant
*Translation*					
*k_tl1_*	5.4			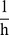	Translation rate constant of *D*
*k_tl2_*	162			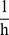	Translation rate constant of *E*
*k_tl3_*	8.1×10^3^			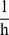	Translation rate constant of *F*
*k_d,F_*	4.8	11.4	4.8	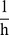	Degradation rate constant of *F*
*k_d_*	0.2			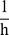	Degradation rate constant of *D* and *E*
*Potassium pools*					
*k_Kdp_*	7.86×10^3^	0.46×10^3^		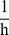	K^+^ uptake rate constant; given that 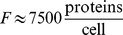 at steady state and cell dry weight is *DW* = 2.8×10^−13^ g, one obtains an estimate of 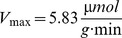 ; in the literature we find 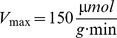 [56]
*K_M,Kdp_*	3.83	4		mM	Half saturation constant of K^+^ uptake; literature value for Kdp: K_M_ = 2 µM
*K_I,Kdp_*	100	0		mM	Inhibition of K^+^ uptake by free K^+^
*V_max,Trk_*	36.5			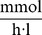	Maximum velocity of K^+^ uptake by Trk
*K_M,Trk_*	0.1			mM	Half saturation constant of K^+^ uptake by Trk
*V_max,Ktr_*	0		400	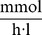	Maximum velocity of K^+^ uptake by KtrAB
*K_M,Ktr_*	0		5×10^−2^	mM	Half saturation constant of K^+^ uptake by KtrAB;
*V_max,lys_*	1×10^−2^	2×10^−2^	1×10^−2^	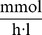	Maximum K^+^ release rate due to cell lysis
*K_M,lys_*	150			mM	Half saturation constant of K^+^ release due to cell lysis
*k_bind_*	8			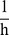	Binding rate constant of free K^+^
*K_M,free_*	250			mM	
*k_diss_*	7.81			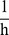	Dissociation rate constant of bound K^+^
*τ*	0	0.35	0	h	“Delay” constant for intracellular K^+^ exchange
*Growth*					
*k_μ,1_*	0.54	0.59	0.54	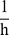	Maximum growth rate
*k_μ,2_*	1.43×10^−3^	1.52×10^−3^	1.57×10^−3^	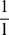	Carrying capacity, inflection point of growth curve
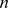	6	1	6	1	Determines maximum steepness of growth curve

The experimental data for the wild type, the RH010 mutant and the RH010/pKT84 mutant cannot be reproduced using a single set of parameters. The Table lists the values of each parameter used to describe the dynamics of each strain.

The final model faithfully reflected the experimental data for *K^+^_free_* at early time points (t = 0–40) after the shift to K^+^ limitation for the RH010 mutant, which showed a nearly 2-fold decrease in *K^+^_free_* compared to the wild type (Fig. 2D and H). At later time points, however, the simulations did not predict the complete depletion of *K^+^_free_* observed experimentally in the mutant. It is important to note that conditions directly after the onset of stress determine the transcription level of *kdpFABC*. Furthermore, an extremely low rate of transport by KdpFA(G345S)BC, or other unknowns which could not be implemented in the model, might influence the *K^+^_free_* pool.

### Simulation of KdpD/KdpE activation in response to varying external K^+^ concentrations

Our mathematical model was designed and calibrated to describe the response of the Kdp system under severe K^+^ limitation. However, we also tested the capacity of the model to qualitatively reflect the response of the *in vivo* system to a broad range of extracellular K^+^ concentrations. With increasing K^+^ availability the constitutive K^+^ uptake system Trk is expected to become more important, and therefore an uptake rate term for this transporter had to be included in the model (see Materials and Methods). Moreover, we assumed that the rate of K^+^ uptake by KdpFABC uptake rate depends on the concentration of free intracellular K^+^ (see Materials and Methods); that is, elevated levels of K^+^ will decrease the uptake velocity. This assumption is reasonable since K^+^ is transported into the cell against its concentration gradient. The values of the parameters introduced with these modifications were chosen empirically to match the experimental data (Table 1).

**Figure 3:**
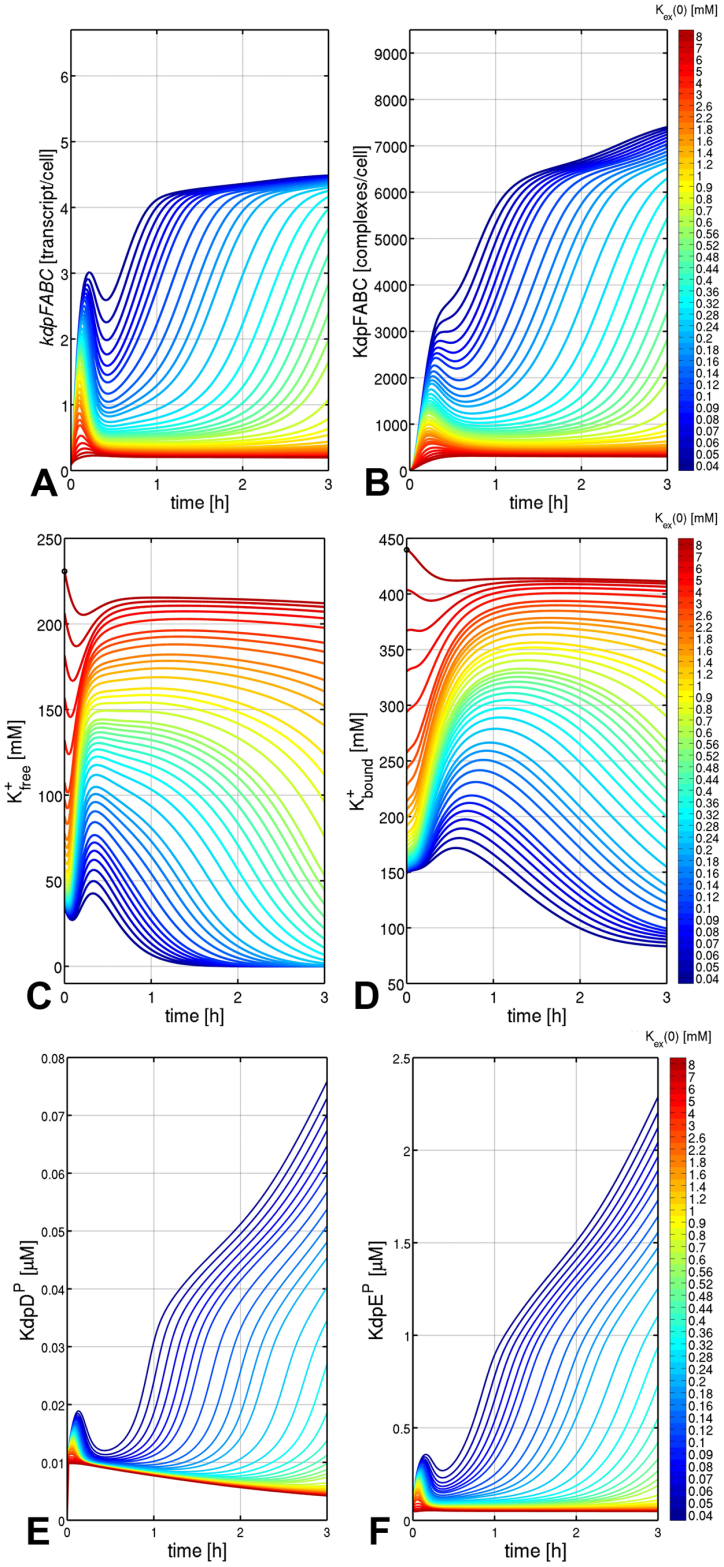
Predicted time-courses of intracellular variables of the Kdp system derived from simulations based on the model for different levels of K^+^ availability. K^+^ availability is determined by the initial concentration of external K^+^ at t = 0 h, *K^+^_ex_*(0). *kdpFABC* transcripts and KdpFABC complexes decrease with increasing *K^+^_ex_* levels (**A, B**). Intracellular K^+^ (free and bound) increases with increasing external K^+^ (**C, D**). Time-courses of phosphorylated KdpD and KdpE are qualitatively very similar to the *kdpFABC* curves (**E, F**).

The model was then used to predict the quantitative behaviour of *kdpFABC* transcripts, KdpFABC complexes and the different K^+^ pools at various levels of *K* ^+^ availability, ranging from severe K^+^ limitation to abundance. Initial levels of extracellular K^+^ (*K^+^_ex_*(0)) were varied from 0.04 to 8 mM. Figure 3 depicts the results of the respective simulations. The transcript numbers (Fig. 3A) followed a non-monotonic time-course over a wide range of *K^+^_ex_* concentrations, whereas numbers of KdpFABC complexes (Fig. 3B) increased monotonically over time.

**Figure 4:**
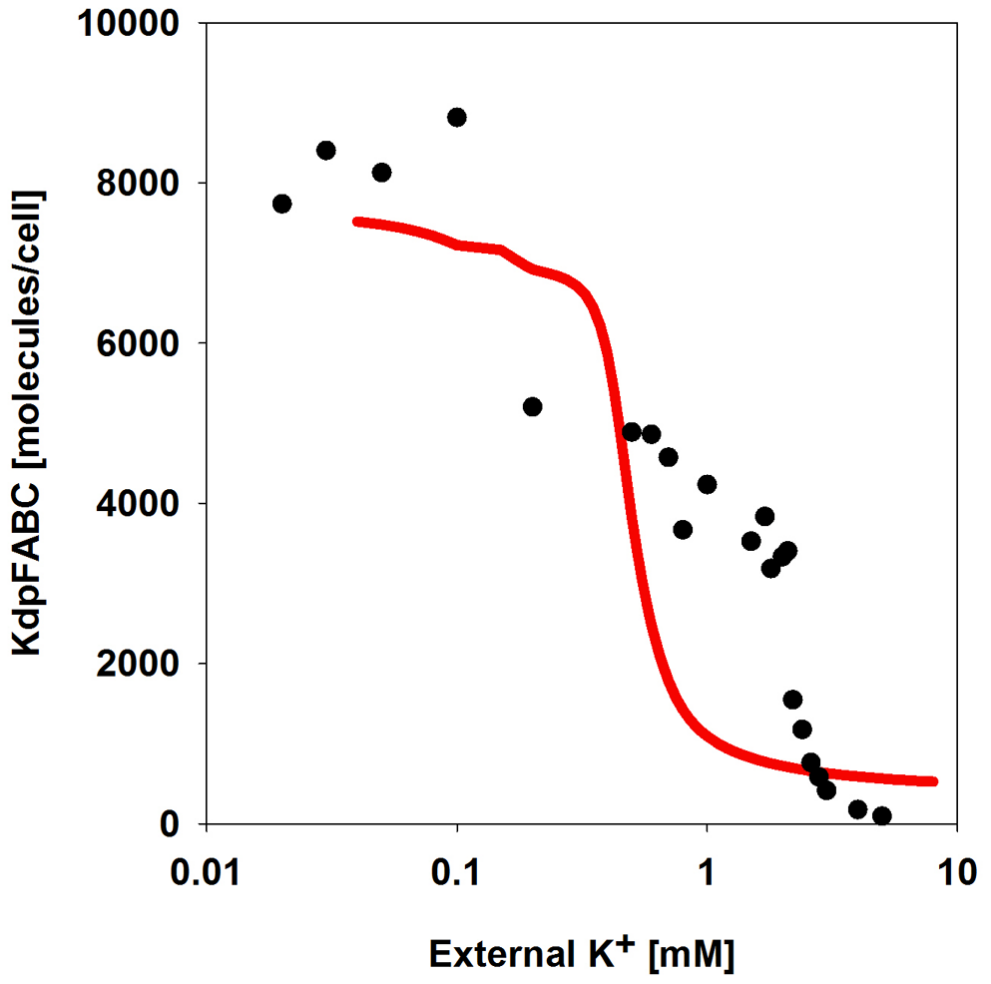
Relationship between the level of KdpFABC at steady state and the initial external K^+^ concentration. Cells were grown in minimal medium containing 10^+^, then shifted in the mid-logarithmic growth phase to the indicated external K^+^ concentrations (40 µM–5.0 mM K^+^), and after 3 h of aerobic incubation at 37°C, cells were harvested, and the level of KdpFABC was determined by quantitative Western blot analysis (•). The red trace depicts the relationship predicted by the model.

The number of KdpFABC molecules predicted to be present 3 h after exposing the cells to the different external K^+^ concentrations (Fig. 3B) was plotted against the external K^+^ concentration (Fig. 4). In parallel, the corresponding experimentally determined numbers were inserted into the same plot (Fig. 4). A clear dependence was found between the number of KdpFABC molecules per cell and the external K^+^ concentration, which reflected the experimentally determined values quite well. Remarkably, the best fit was achieved at severe K^+^ limitation (0.02 to 0.1 mM K^+^), whereas under moderate (0.1 to 2.1 mM) or higher K^+^ concentrations (>2.1 mM), the model captured the decrease in KdpFABC production qualitatively but not quantitatively (Fig. 4).

We also predicted the time-courses of free and bound K^+^ (Fig. 3C, D). Under extreme K^+^ limitation, uptake of the residual external K^+^ initially out-weights dilution by cell growth. Then the residual cell growth becomes the dominant factor, while at high external K^+^ concentrations (1–8 mM), K^+^ uptake constantly compensates for dilution of the internal K^+^ pools by cell growth.

The model was also used to simulate and predict the concentrations of phosphorylated KdpD and KdpE at the various external K^+^ concentrations. Since experimental determination of KdpD and KdpE phosphorylation kinetics *in vivo* is not possible thus far, the simulations can be seen as virtual measurements. Both KdpD-P and KdpE-P exhibited non-monotonic time-courses for all initial *K^+^_ex_* concentrations (Fig. 3E, F).

## Discussion

In natural environments bacteria are often exposed to long-lasting periods of stress. Hence, not only must they be capable of adapting rapidly to changing conditions, they must also be able to cope with persisting stress conditions. With this consideration in mind, we quantitatively analysed and simulated the dynamics of the *E. coli* Kdp system over a period of 3 h after activation.

When *E. coli* is exposed to limiting K^+^ concentrations, the KdpD/KdpE signalling cascade is instantly activated and, consequently, *kdpFABC* transcription is rapidly induced. After a lag period required for its synthesis and assembly, the KdpFABC complex then mediates high-affinity K^+^ uptake. Thus *kdpFABC* expression was turned on immediately after shifting wild type *E. coli* cells into medium containing a very low level of K^+^ (40 µM), reached a peak in less than 20 min, and then declined before rising again at 30 min. This transient dip points to negative feedback regulation of *kdpFABC* transcription, which was predicted in our earlier *in silico* and *in vivo* studies, and was also observed in an independent study [32], [36]. Negative feedback regulation is frequently employed during stress-dependent processes in bacteria to save energy for mRNA synthesis [44]. By monitoring our cultures for a longer time period, we found that shortly after the first induction peak, the concentration of free intracellular K^+^ falls significantly, and the cells essentially run out of K^+^ once more. It is important to note that this loss of K^+^ is attributed to the onset of growth. Consequently, the KdpD/KdpE signalling cascade and *kdpFABC* transcription are activated for a second time. At this stage, the level of *kdpFABC* expression begins to rise again to meet the cell's significantly increased need for K^+^. Thereafter (t = 60 min) a steady state is attained, marked by continued transcription of *kdpFABC* and synthesis (and very probably turnover) of KdpFABC, cell division and cell lysis, with concomitant release and re-uptake of K^+^. Note that, over the whole time-course, the extracellular K^+^ concentration decreased at a constant rate, and was clearly uncoupled from KdpFABC production and hence from KdpD/KdpE activation.

The observed pattern of KdpFABC production, and the known inhibitory effect of K^+^ on KdpD/KdpE phosphorylation in vitro [29]–[31], provide clues to the nature of the primary stimulus for KdpD phosphorylation. It is conceivable that due to the shift of cells into a low-K^+^ environment, the ion's inhibitory effect on the phosphorylation of KdpD/KdpE is relieved. Specifically, according to our simulations, inhibition of the phosphatase activity of KdpD is required to keep KdpE in the phosphorylated state and enable high-level production of KdpFABC, which in turn increases the amount of free K^+^ in the cell. Our calculations indicated that this increase in the concentration of free intracellular K^+^ enhances KdpD phosphatase activity and thus reduces the concentration of phospho-KdpE. This is the first indication of an inverse correlation between KdpD/KdpE phosphorylation and the concentration of free intracellular K^+^.

The hypothesis that the intracellular K^+^ concentration modulates KdpD/KdpE phosphorylation is further supported by our finding that no hint of negative feedback regulation is observed when the KdpFABC transport-defective *E. coli* mutant RH010 is subjected to similar K^+^ limitation. Moreover, the expression profiles in the RH010 mutant also argue against an effect of the extracellular K^+^ concentration on KdpD/KdpE activation under these conditions. Thus, although - due to the defect in K^+^ uptake by the mutant KdpFABC complex - the extracellular K^+^ concentration declines more slowly than in case of the wild type, levels of *kdpFABC* transcripts rise to far higher levels. Notably, *trans*-complementation of this *E. coli* mutant by the high-affinity KtrAB K^+^ transporter from *V. alginolyticus* reduced *kdpFABC* expression to a level comparable to that in wild type. The experimentally determined and theoretically modelled data for the non-complemented mutant strongly support the notion that the intracellular K^+^ concentration is an important modulator of KdpD/KdpE activities under conditions of severe K^+^ limitation. Concomitantly, these results throw light on the mode of negative feedback regulation [36] by which the uptake of K^+^ by the KdpFABC complex down-regulates its own expression. Regulation of KdpD/KdpE phosphorylation by the intracellular K^+^ concentration is in line with earlier observations which indicated that both extracellular Cs^+^ and low pH significantly reduce the availability of free intracellular K^+^ and lead to induction of *kdpFABC* expression [27], [28].

However, *kdpFABC* expression, and therefore KdpFABC production, is not only induced at very low external K^+^ concentrations (below 0.1 mM), but gradually increases at moderate K^+^-limiting conditions (2 to 0.1 mM) [22], [23], [32]. Our simulations very well captured this phenomenon, too (Fig. 3A, B, Fig. 4). Though, the simulations perfectly reproduced the experimental data at K^+^ concentrations below 0.1 mM, but deviated from them under moderately K^+^-limiting conditions (0.1 to 2 mM) and non-stress conditions (above 2.1 mM) (Fig. 4).

Therefore, it is suggested that the internal K^+^ pool predominantly acts as primary stimulus under extreme K^+^ limitation, and that external K^+^ influences KdpD/KdpE activation under moderate stress conditions, when a significant drop in the intracellular K^+^ concentration is unlikely (Figs. 3C and 3D). This proposal is in accord with recent findings by Altendorf and coworkers, who suggest that KdpD senses the external K^+^ concentration as one stimulus [32]. Nevertheless, this hypothesis needs further experimentation since evidence for a periplasmic K^+^-binding site is lacking, and it is unclear whether external K^+^ influences the kinase or the phosphatase activity of KdpD.

During modelling it emerged that the response of the RH010 mutant that produces an inactive KdpFABC was hampered by the fact that the ratio of transcript to protein at steady-state differed from the values for the wild type strain. By means of sensitivity analysis, one parameter (*K*) could be identified which lumps together several kinetic constants for transcription initiation. After re-estimation the accuracy could be improved. It is likely that the Kdp system is not only controlled at the transcriptional level, but also at the post-translational level by proteolysis, and at the activity level [26]. Whether post-translational regulation is linked to K^+^ is unclear.

In summary, our study reveals that the tight interplay between theory and experiment greatly helps to improve our understanding of bacterial sensing and signalling pathways. Moreover, it also highlights how important it is to record the time-dependent input/output ratios of these systems. Lastly, this is one of the rare examples demonstrating the bacterial dynamics during the management of prolonged stress.

## Materials and Methods

### Materials

[γ-^32^P]ATP and [^35^S]methionine were purchased from GE Healthcare (Munich), and ^3^H_2_O and [^14^C]sucrose were obtained from Biotrend (Cologne). SYBR Green Mix was from BioRad (Munich), the DyNAmo™ cDNA Synthesis Kit and Protein A magnetic beads were from New England Biolabs (Frankfurt am Main). Goat anti-(rabbit IgG)-alkaline phosphatase was obtained from Biomol (Hamburg). Purified KdpFABC protein was supplied by Marc Bramkamp (Osnabrück University). RNase-free deoxyribonuclease I was from Fermentas (St. Leon-Rot), and silicone oil (DC550) was from Serva (Heidelberg). All other reagents were reagent grade and obtained from commercial sources.

### Strains and plasmids

*E. coli* strains MG655 (wild type K-12 strain) [45], MG1655 *rpsL150* [40], and *E. coli* RH010 (MG1655 *rpsL150 kdpA4*) [40] were used for these studies. *E. coli* RH010 is characterized by a chromosomal point mutation in *kdpA* (G1033A), which leads to the replacement of the glycine at position 345 in KdpA by serine, and results in a KdpFABC complex that is defective in K^+^ transport. Plasmid pKT84 [42] encodes *ktrAB* under the control of its native promoter.

### Measurement of *kdpFABC* expression by quantitative RT-PCR

Transcription of *kdpFABC* under K^+^ limitation was monitored by quantitative RT-PCR (qRT-PCR). *E. coli* MG1655, *E. coli* MG1655 *rpsL150* and *E. coli* RH010 (MG1655 *rpsL150 kdpA4*) were aerobically grown in phosphate-buffered minimal medium containing 10 mM K^+^ [46] until the mid-exponential growth phase and then shifted to fresh, pre-warmed medium containing 40 µM (K^+^ limitation) by filtration. Cultivation was continued at 37°C. For cultivation of *E. coli* RH010 pKT84, the medium was supplemented with 100 µg×ml^−1^ ampicillin. At the times indicated, samples were taken from the cultures, and total RNA was isolated using acidic phenol/chloroform [47]. Contaminating genomic DNA was removed by DNase digestion (DNase I, RNase-free, Fermentas). Aliquots (4 µg) of total RNA were subjected to reverse transcription using the RevertAid™ First Strand cDNA Synthesis Kit (Fermentas) and an oligo (dt)_18_ primer, and the resulting cDNA was used for subsequent quantitative real-time PCR. qRT-PCR was conducted on an iQ5 Multicolor Real-Time PCR Detection System (BioRad) using the Maxim SYBR Green/ROX qPCR Master Mix (BioRad) and analysed with the iQ5 Optical System Software (BioRad). *kdpA* was amplified by qPCR using primers 5′-CCAACCGCACTGACCAACTTC-3′ and 5′-TCGCCCATCACTTCACCAAAG-3′. *recA* was used as the reference gene. Prior to qPCR procedure, all samples were heated for 3 min at 95°C, and then taken through 40 cycles of 10 s at 95°C and 30 s at 65°C. Binding of the primers for the qPCR and amplification of the appropriate single PCR product corresponding to the *kdpA* gene were checked by melting-curve analysis and gel electrophoreses. All reactions were performed on three biological replicates and the transcript amount was calculated using the ΔΔC^t^ method [48].

### Determination of extracellular and intracellular K^+^ concentrations

K^+^ concentrations were determined by atomic absorption spectroscopy [49]. Briefly, 1-ml samples taken from a growing *E. coli* culture were centrifuged through 200 µl silicone oil (DC550; 1∶10 diluted with hexadecane) for 2 min at 13,000 rpm. The K^+^ content of the cell pellets and the supernatants were determined in an atomic absorption spectrometer (Varian AA240 Spectroscopy Instrument, Agilent Technologies, Böblingen). To determine the fraction of bound and freely diffusible K^+^, duplicate 0.5-ml samples were collected and centrifuged. Cell pellets were resuspended in either 0.5 ml medium (total K^+^) or 0.5 ml ddH_2_O (bound K^+^) [49]. After centrifugation through silicone oil, the K^+^ content of the cell pellets was determined by atomic absorption spectrometry. The fraction of free K^+^ is defined as the difference between the total K^+^ content and the bound K^+^ content. The intracellular concentrations were calculated by taking the number of cells per sample and the cytoplasmic volumes into account. Since cell volumes were found to remain more or less constant during the experiments (Table S1), an average value of 8.12×10^−16^ l per cell was used in all calculations.

### Determination of cytoplasmic volume

The size of the cytoplasmic water space can be used as a proxy for the cytoplasmic volume [50], and was determined as described by Altendorf and coworkers [23]. Since [^14^C]sucrose cannot be metabolized by *E. coli* MG1655 (as shown previously) and therefore does not enter the cytoplasm, in contrast to ^3^H_2_O, the cytoplasmic volume could be calculated by determining the difference between their distributions, taking their nuclide content into account. Briefly, ^3^H_2_O (1 mCi/ml) and [^14^C]sucrose (0.1 mCi/ml, glucose-free, ubiquitously labelled) were added to 1.3-ml samples of growing *E. coli* cells. After 1 min, cells were centrifuged for 2 min through a 200-µl silicone oil layer of appropriate density (see above). The cell pellets were separated from the supernatants, and cells were lysed by treatment with 1 ml 0.4 M NaOH at 62°C for 1 h. The radioactivity of the two labelled compounds in the cell pellet was quantified simultaneously by liquid scintillation counting. The dual-label protocol was used so the original counts per minute of each nuclide could be converted into disintegrations per minute (dpm) taking the overlap of the energy distribution spectra of the two nuclides into account. For quantification, the radioactivity present in 50 µl of supernatant was also determined. As ^3^H_2_O penetrates into every part of the cell, the total cellular water space including the extracellular water layer was calculated as follows: (^3^H dpm of the pellet) ×50 µl/(^3^H dpm of 50 µl supernatant). Since [^14^C]sucrose is only excluded from the cytoplasm, the volume of the extracellular water layer plus the periplasmic space was calculated as follows: (^14^C dpm of the pellet) ×50 µl/(^14^C dpm of 50 µl supernatant).

The difference between the space occupied by ^3^H_2_O and the space occupied by [^14^C]sucrose gives the volume of cytoplasmic water, which includes all water layers bound to biological surfaces, the so-called bound water [51], [52], as well as the freely diffusing water, the so-called bulk water. The water space of the cytoplasm is referred to as the cytoplasmic volume, which does not encompass the volume of all macromolecules in the cytoplasm. The concentration of cytoplasmic solutes was calculated on the basis of the cytoplasmic volume.

### Determination of KdpFABC production by quantitative Western blot analysis

Levels of KdpFABC in *E. coli* strains were determined by quantitative Western blot analysis. The respective *E. coli* strains were grown as described above. At the time points indicated, aliquots of the cultures were removed and the cells were collected by centrifugation, resuspended in SDS sample buffer and subjected to SDS-PAGE [53]. Known concentrations of purified KdpFABC were used to obtain a standard curve, and 0.1 µg of purified KdpFABC was also loaded onto each gel as a standard. Coomassie Blue staining of separate gels verified loading of equal amounts of protein. Proteins were then electro-blotted onto a nitrocellulose membrane, and the blots were blocked with 5% (w/v) skim milk in buffer A (10 mM Tris/HCl pH 7.4, 0.15 M NaCl) for 1 h. Anti-KdpB antibody [30] was added at a final dilution of 1∶10,000 and incubation was continued for 1 h. After washing with buffer A, goat anti-(rabbit IgG) conjugated with alkaline phosphatase was added in a final dilution of 1∶2,500, and incubation was continued for 1 h. After washing thoroughly, blots were developed with substrate solution [50 mM NaCO_3_, pH 9.5, 0.01% (w/v) nitro-blue-tetrazolium, 5 mg/ml 5-bromo-4-chloro-3-indolylphosphate]. Blots were scanned at high resolution in 256 grey scales and imported as TIF files into ImageQuant 5.0, and the amount of KdpB was quantified by comparison with the standard curve.

### *In vivo* protein stability assay

The stability of KdpFABC under K^+^ limitation was analysed *in vivo* in *E. coli* MG1655 *rpsL150* and *E. coli* RH010 (MG1655 *rpsL150 kdpA4*) by labelling the complex with [^35^S]methionine. Cells were grown to exponential phase and shifted to K^+^ limitation as described above. After 10 min, 2.5 µCi/ml (final concentration; 1000 Ci/mmol) [^35^S]methionine was added, and cells were incubated for an additional 10 min to allow uptake of label into *de novo* synthesized proteins, in this case predominantly the subunits of the KdpFABC. Then, non-radioactive methionine was added in excess (2 mM final concentration) to block further radiolabeling of nascent proteins. After different times 1-ml culture aliquots were collected, and immediately frozen in liquid nitrogen to stop protein biosynthesis. Before detection of radiolabeled KdpFABC, the protein was concentrated using immunomagnetic separation. For this purpose, cells were sonified (3×30 sec, interrupted by 30-sec breaks, 50% magnitude) and proteins were solubilized with 1.5% (w/v) *n*-dodecyl-β-D-maltoside. αKdpFABC antiserum [30] containing 5 µg protein (antibodies) was added, and samples were incubated with gentle agitation at 4°C for 1 h. Then, Protein-A-coupled magnetic beads with a total binding capacity of 10 µg human IgG were added (25 µl), and samples were further incubated at 4°C for 1 h. Beads were recovered by applying a magnetic field, washed three times with buffer [10 mM Tris/HCl pH 7.5, 150 mM NaCl, 1 mM EDTA, 1 mM EGTA, 0.2 mM PMSF, 1.5% (w/v) *n*-dodecyl-β-D-maltoside], and proteins were finally removed from the beads by addition of 30 µl 3-fold concentrated SDS sample buffer [53] containing 4 M urea. Samples including a purified KdpFABC that had also been radiolabelled with ^35^S, were then subjected to SDS-PAGE [53], stained with Coomassie Blue, and gels were dried. Radiolabeled KdpFABC was detected by autoradiography of the stained and dried gel and quantified by comparison with the signal for the standard.

### Mathematical model for the Kdp system

The mathematical model of the Kdp system [36] is composed of two modules: the first module captures the interactions between KdpD and KdpE, the second describes the transcription of the target regulon *kdpFABCDE* and the synthesis of its protein products. The first module comprises the rate equations for autophosphorylation (R1) of the sensor kinase KdpD [*D*], transfer of the phosphoryl group (R2) from KdpD-P [*D^P^*] to the response regulator KdpE [*E*], and the dephosphorylation (R3) of free KdpE-P [*E^P^_f_*] resulting in inorganic phosphate [P_i_]:
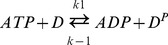
(R1)

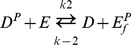
(R2)

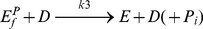
(R3)In addition, the module describes the initiation of transcription, which depends on the interaction of polymerase and KdpE proteins with free DNA. This process can be described by the following set of reactions
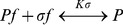
(R4a)

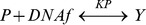
(R4b)

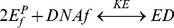
(R4c)

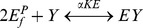
(R4d)

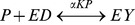
(R4e)These reactions comprise the binding of free polymerase [*P_f_*] to free sigma factor [*σ_f_*] (R4a), the binding of the polymerase complex [*P*] to promoter DNA [*DNA_f_*] ((R4b)+(R4e)) and the binding of two free phosphorylated KdpE response regulator proteins [*E^P^_f_*] to their cognate binding site in the DNA ((R4c)+(R4d)). The order of binding of polymerase and response regulator is mutually independent; however the second reactant binds with a higher affinity, represented by the factor *α*. The parameters *K_i_ (i = σ,P,E)* denote the equilibrium dissociation constants *K_i_ = k_-i_/k_i_*. Reactions (R4a)-(R4e) are assumed to be very fast in comparison to protein synthesis. Therefore the rapid equilibrium approach was applied to each of the reactions, leading to a set of algebraic equations that describe the protein complexes as functions of the protein concentrations and the reaction constants:
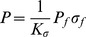


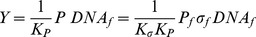


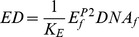


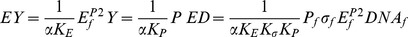
We obtain the following conservation relations for the total concentration of DNA
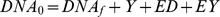
and the total concentration of phosphorylated response regulator
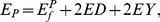
with
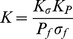
(Since experimental quantification of *P_f_* and *σ_f_* is almost impossible, both variables are considered to be constant and are combined in a single parameter *K*, together with the dissociation constants *K_σ_* and *K_P_*).

Thus, the mathematical description of the first module comprises two differential equations for *D^P^* and *E^P^* (total amount of phosphorylated response regulator, free and DNA-bound), two algebraic equations to calculate the amounts of free DNA *DNA_f_*, and the unbound response regulator *E^P^_f_*, and two algebraic equations to determine the amount of unphosphorylated sensor kinase *D* and response regulator *E*:
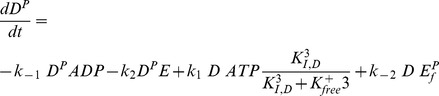


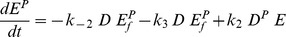


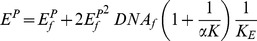


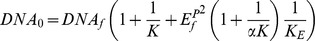


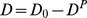


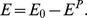

*D*_0_ and *E*_0_ are the total concentrations of the sensor kinase KdpD and the response regulator KdpE, respectively. The balance equations for these variables will be derived in the following. The concentration of *DNA*_0_ is always taken to be constant.

To describe the overall module up to the level of KdpFABC [*F*] production, the model was extended to include a second module comprising equations for the transcript dynamics (reflecting synthesis and stability of the mRNA), and the dynamics of the proteins KdpFABC, KdpD and KdpE:
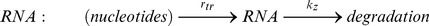


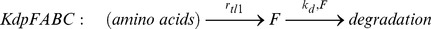


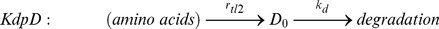


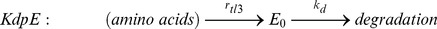
Since it is assumed that the concentration of nucleotides and amino acids is not limiting, the rate laws *r_tr_* and *r_tli_* (i = 1, 2, 3) do not depend on the monomer concentration.

Using the relations derived for the first module, transcription efficiency (i.e. relative promoter occupancy) is calculated as
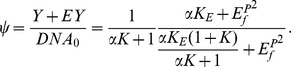
Following a method established earlier [54], the rate of mRNA synthesis *r_tr_* is then calculated with the following equation:
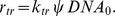
Thus, the differential equations for transcript and protein synthesis read:
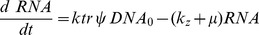


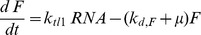


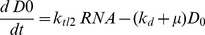


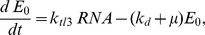
where *F* represents the K^+^ uptake system KdpFABC and *μ* the specific growth rate of the cells. The rate of degradation of KdpFABC is chosen to be a linear function of the protein concentration, i.e. *r_deg,F_ = k_d,F_ F*. This choice is discussed in more detail in the Results section in relation to the putative controlled proteolysis.

In the previously published model [36] the feedback effect of KdpFABC on the two-component system and its dependence on K^+^ concentration was described by a black box approach. It was assumed that an increase in the intracellular K^+^ concentration mediated by KdpFABC-dependent K^+^ uptake should promote the dephosphorylation of *E^P^_f_* (via parameter *k_3_*). This was described by the following equation
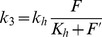
where *k_h_* and *K_h_* represent adjustable parameters.

In order to turn the black box model into more of a grey box, which allows a better understanding of the mechanism of K^+^ uptake by *E. coli*, detailed mass balance equations for intra- and extracellular K^+^ concentrations were included. Intracellular K^+^ is assumed to exist in two pools: One fraction is bound to macromolecules while the other is assumed to be freely diffusible in the cell.

The pools of extracellular, and free and bound intracellular, K^+^ are linked with each other: Extracellular K^+^ is taken up by viable cells and added to the pool of the free intracellular K^+^. The pools of free and bound K^+^ are connected by binding to/dissociation from macromolecules. Due to the lack of direct quantitative information on these processes, the respective kinetics was empirically chosen as
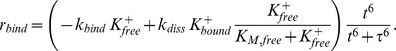
This formulation ensures that free and bound K^+^ equilibrate at high K^+^ availability. The artificial delay term 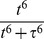 was introduced to account for the dynamics of the free and bound K^+^ fractions in the RH010 mutant (see Fig. 2), where there is almost no exchange between free and bound K^+^ for the first 20 min. Additionally, dead cells are assumed to release K^+^ (free and bound) into the medium.

Transport processes between extracellular space and cytoplasm are modeled using a Michaelis-Menten rate law
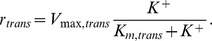
In order to account for the time-dependent variations in the number of KdpFABC complexes, the *V_max_* value of the uptake rate is a function of the KdpFABC concentration:
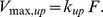
Depending on the respective strain, K^+^ is taken up by up to three transporters: i) In the wild type, KdpFABC and Trk are present, ii) in the RH010 mutant, a KdpFABC with reduced transport capacity and Trk are present, and iii) in the RH010 mutant carrying plasmid pKT84, a KdpFABC with reduced transport activity, Trk and the KtrAB transporter are all present. Therefore, in its most general formulation, the model comprises three different K^+^ uptake terms, one for each transporter. Besides that, there is one term to describe K^+^ efflux due to cell lysis.

In addition, we have to take into account that the extracellular K^+^ concentration is normalized with respect to the medium volume that is seen by all cells, whereas the intracellular concentrations are normalized with respect to the volume of a single cell. Therefore, in the balance equation for the extracellular K^+^ concentration, both the K^+^ uptake rate and the efflux rate due to cell lysis are proportional to the total volume of all cells. Taken together, these considerations led to a set of four additional differential equations that characterize K^+^ uptake:
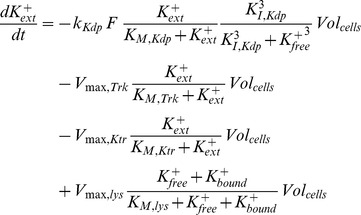


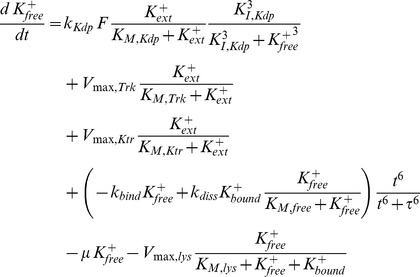


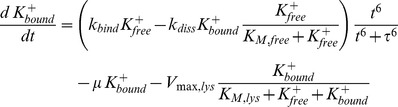


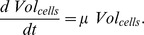
It should be noted that it is not possible to distinguish between the free and bound K^+^ balances in viable and dead cells. Therefore, in the model the average cell takes up and releases K^+^ at the same time. Due to K^+^ limiting conditions, the growth rate *μ* is not constant and varies over time. This variable was modelled using the approach
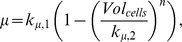
so that the solution of the differential equation *dVol_cells_/dt = μVol_cells_* corresponds to the generalized logistic function, an asymmetric sigmoid curve which provides a convenient approach for the modelling of growth of different organisms [55].

There are several alternative ways of modelling the influence of K^+^ on the KdpD/KdpE two-component system. In order to account for the non-monotonic dynamics of the *kdpFABC* transcript, K^+^ could either inhibit the autophosphorylation of KdpD or amplify/enhance the dephosphorylation of KdpE-P, or both. We modelled the possible inhibiting effect of K^+^ on the kinase activity of KdpD by taking
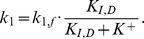
The enhancing effect of K^+^ on the phosphatase activity of KdpD was modelled by taking
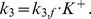
In the final model, we assumed that an increase in the free intracellular K^+^ concentration mediated by K^+^ uptake through KdpFABC should increase the dephosphorylation of *E^P^_f_* (via parameter *k_3_*) so that
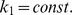
and
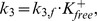
where *k_3_,_f_* represents an adjustable parameter.

For the analysis of proteolysis, the degradation of the corresponding protein [*P*] was modelled using the following differential equation
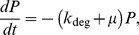
where *k_deg_* is the degradation time constant. If protein degradation is actively controlled, *k_deg_* is expected to vary under different experimental conditions. The specific growth rate *μ* is also time dependent; therefore it is determined using the empirical equation given above.

### Model calibration

Prior to parameter estimation, parameter sensitivities and correlations needed to be analysed in order to detect potential sources of identifiability problems. Parameters that show very little sensitivity compared to others, as well as correlations among parameters (which would indicate that only combinations of parameters, rather than single ones, can be identified), can lead to far-from-optimal results (the theoretical foundations of this topic are beyond the scope of this study, therefore we refer the reader to [43] and the references therein).

Several parameters that determine the dynamics of the two-component system (*k_-1_, k_2_, k_-2_*) showed very little sensitivity and were highly correlated with each other. Therefore, these parameters are virtually unidentifiable and could be set to nominal values, and were therefore excluded from parameter estimation. The parameters describing the release of K^+^ due to cell lysis and the synthesis of the proteins KdpD and KdpE also showed low sensitivities. In addition, we found high correlations among the parameters describing the transcription of *kdpFABC*.

In order to describe and reproduce the experimental data for the RH010 mutant, the wild type model was used, albeit with appropriate adjustments of several parameters. To account for the lower rate of K^+^ uptake, the value of the parameter *k_Kdp_* was changed (Table 1). Furthermore, the increase in transcripts/cell in this mutant was greater than in the wild type. By means of sensitivity analysis of the model this observation could theoretically be explained by changes in each of the parameters that describe transcription efficiency and transcript synthesis, i.e. *α, K_E_, K, DNA*_0_ *and k_tr_*. Sensitivity analysis revealed that parameter *K* has a strong impact on the steady-state level; therefore, this parameter was adjusted accordingly. *K* is a lumped/aggregated parameter that contains several variables, namely the (unknown) concentrations of the sigma factor and polymerase, as well as the equilibrium constants for the reactions in which these two variables are involved

The RH010 mutant was also complemented in trans with the high-affinity K^+^ transporter KtrAB from Vibrio alginolyticus [42] and analysed as described before (Fig. 2J–M). The degree of induction of kdpFABC in the complemented mutant was comparable to that in the wild type (Fig. 2J–M). However, the dynamics of transcription was qualitatively similar to that observed for the RH010 mutant, albeit over a more restricted dynamic range. The time-courses of the extra- and intracellular K^+^ concentrations showed the same qualitative behaviour as in the wild type. However, the intracellular concentrations of free and bound K^+^ were approximately 30% lower than those measured for the wild type. Hence, the mathematical model of the wild type was modified to match this situation. We added another uptake term
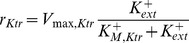
to describe the uptake by KtrAB, and the parameters *V_max,Ktr_* and *K_M,Ktr_* were adjusted in accordance with the measured K^+^ data. With these modifications, the model was found to reproduce the experimental findings quite well.

## Supporting Information

Figure S1 **Proteolysis controls appropriate KdpFABC level.** *E. coli* MG1655 *rpsL150* (wild type) and *E. coli* RH010 were cultivated in phosphate buffered minimal medium containing 10 mM K^+^ up to the mid-logarithmic growth phase, exposed to extreme K^+^ limitation (0.04 mM K^+^) for 10 min to activate *kdpFABC* expression before proteins were labelled with ^35^S-methionine. Labelling of *de novo* synthesized proteins was quenched by adding an excess of non-labelled methionine after 10 min. Protein turnover is determined by the reduction of the labelled protein complex over the time. At different times, samples were taken, immunoprecipitated with αKdpFABC antiserum, subjected to SDS-PAGE, and the amount of KdpFABC was quantified from the autoradiographies of the gels. The data represent one of three independently performed characteristic experiments. Half-life was determined to be 36.7 min for wild type KdpFABC and 28.4 min KdpFA(G345S)BC.
(TIF)

Table S1 **Total and viable cell numbers and cytoplasmic volume after exposure of** ***E. coli*** **to K^+^ limitation.** *E. coli* MG1655 cells were grown in minimal medium containing 10 mM K^+^ to the mid-logarithmic growth phase and shifted to K^+^ limitation (40 µM K^+^). At the indicated times, cells were harvested, and the total and viable cell numbers as well as the cytoplasmic volume of the cells were determined as described in Materials and Methods. The values represent average values of at least three independent experiments. Standard deviations were below 5%. n.d., not determined.
(DOCX)

## References

[1] Epstein W (2003) The roles and regulation of potassium in bacteria. Prog Nucleic Acid Res Mol Biol 75: 293–320. 14604015 10.1016/s0079-6603(03)75008-9

[2] Booth IR (1985) Regulation of cytoplasmic pH in bacteria. Microbiol Rev 49: 359–378. 3912654 10.1128/mr.49.4.359-378.1985 PMC373043

[3] Suelter CH (1970) Enzymes activated by monovalent cations. Science 168: 789–795. 5444055 10.1126/science.168.3933.789

[4] Rübenhagen R, Morbach S, Krämer R (2001) The osmoreactive betaine carrier BetP from *Corynebacterium glutamicum* is a sensor for cytoplasmic K^+^ . EMBO J 20: 5412–5420 10.1093/emboj/20.19.5412 11574473 PMC125657

[5] Giaever HM, Styrvold OB, Kaasen I, Strøm AR (1988) Biochemical and genetic characterization of osmoregulatory trehalose synthesis in *Escherichia coli* . J Bacteriol 170: 2841–2849. 3131312 10.1128/jb.170.6.2841-2849.1988 PMC211211

[6] Sutherland L, Cairney J, Elmore MJ, Booth IR, Higgins CF (1986) Osmotic regulation of transcription: induction of the *proU* betaine transport gene is dependent on accumulation of intracellular potassium. J Bacteriol 168: 805–814. 3536861 10.1128/jb.168.2.805-814.1986 PMC213556

[7] Lee SJ, Gralla JD (2004) Osmo-regulation of bacterial transcription via poised RNA polymerase. Mol Cell 14: 153–162. 15099515 10.1016/s1097-2765(04)00202-3

[8] Nissen P, Hansen J, Ban N, Moore PB, Steitz TA (2000) The structural basis of ribosome activity in peptide bond synthesis. Science 289: 920–930. 10937990 10.1126/science.289.5481.920

[9] Csonka LN, Hanson AD (1991) Prokaryotic osmoregulation: genetics and physiology. Annu Rev Microbiol 45: 569–606 10.1146/annurev.mi.45.100191.003033 1741624

[10] Palleros DR, Reid KL, Shi L, Welch WJ, Fink AL (1993) ATP-induced protein-Hsp70 complex dissociation requires K^+^ but not ATP hydrolysis. Nature 365: 664–666 10.1038/365664a0 8413631

[11] Schlösser A, Meldorf M, Stumpe S, Bakker EP, Epstein W (1995) TrkH and its homolog, TrkG, determine the specificity and kinetics of cation transport by the Trk system of *Escherichia coli* . J Bacteriol 177: 1908–1910. 7896723 10.1128/jb.177.7.1908-1910.1995 PMC176828

[12] Stumpe S, Bakker EP (1997) Requirement of a large K^+^-uptake capacity and of extracytoplasmic protease activity for protamine resistance of *Escherichia coli* . Arch Microbiol 167: 126–136. 9133319

[13] Ballal A, Basu B, Apte SK (2007) The Kdp-ATPase system and its regulation. J Biosci 32: 559–568. 17536175 10.1007/s12038-007-0055-7

[14] Altendorf K, Siebers A, Epstein W (1992) The Kdp-ATPase of *Escherichia coli* . Ann N Y Acad Sci 671: 228–243. 1288322 10.1111/j.1749-6632.1992.tb43799.x

[15] Laimins LA, Rhoads DB, Epstein W (1981) Osmotic control of *kdp* operon expression in *Escherichia coli* . Proc Natl Acad Sci USA 78: 464–468. 6787588 10.1073/pnas.78.1.464 PMC319074

[16] Walderhaug MO, Polarek JW, Voelkner P, Daniel JM, Hesse JE, et al (1992) KdpD and KdpE, proteins that control expression of the *kdpABC* operon, are members of the two-component sensor-effector class of regulators. J Bacteriol 174: 2152–2159. 1532388 10.1128/jb.174.7.2152-2159.1992 PMC205833

[17] Heermann R, Jung K (2010) The complexity of the “simple” two-component system KdpD/KdpE in *Escherichia coli* . FEMS Microbiol Lett 304: 97–106 10.1111/j.1574-6968.2010.01906.x 20146748

[18] Heermann R, Jung K (2012) K^+^ supply, osmotic stress and the KdpD/KdpE two-component system. In: Gross R, Beier D, editors. Two-component systems in bacteria. Norwich, UK: Caister Academic Press. pp. 181–198.

[19] Voelkner P, Puppe W, Altendorf K (1993) Characterization of the KdpD protein, the sensor kinase of the K^+^-translocating Kdp system of *Escherichia coli* . Eur J Biochem 217: 1019–1026. 8223625 10.1111/j.1432-1033.1993.tb18333.x

[20] Sugiura A, Nakashima K, Tanaka K, Mizuno T (1992) Clarification of the structural and functional features of the osmoregulated *kdp* operon of *Escherichia coli* . Mol Microbiol 6: 1769–1776. 1630316 10.1111/j.1365-2958.1992.tb01349.x

[21] Jung K, Tjaden B, Altendorf K (1997) Purification, reconstitution, and characterization of KdpD, the turgor sensor of *Escherichia coli* . J Biol Chem 272: 10847–10852. 9099740 10.1074/jbc.272.16.10847

[22] Malli R, Epstein W (1998) Expression of the Kdp-ATPase is consistent with regulation by turgor pressure. J Bacteriol 180: 5102–5108. 9748442 10.1128/jb.180.19.5102-5108.1998 PMC107545

[23] Hamann K, Zimmann P, Altendorf K (2008) Reduction of turgor is not the stimulus for the sensor kinase KdpD of *Escherichia coli* . J Bacteriol 190: 2360–2367 10.1128/JB.01635-07 18245296 PMC2293187

[24] Asha H, Gowrishankar J (1993) Regulation of *kdp* operon expression in *Escherichia coli*: evidence against turgor as signal for transcriptional control. J Bacteriol 175: 4528–4537. 8331081 10.1128/jb.175.14.4528-4537.1993 PMC204895

[25] Frymier JS, Reed TD, Fletcher SA, Csonka LN (1997) Characterization of transcriptional regulation of the *kdp* operon of *Salmonella typhimurium* . J Bacteriol 179: 3061–3063. 9139930 10.1128/jb.179.9.3061-3063.1997 PMC179076

[26] Roe AJ, McLaggan D, O'Byrne CP, Booth IR (2000) Rapid inactivation of the *Escherichia coli* Kdp K^+^ uptake system by high potassium concentrations. Mol Microbiol 35: 1235–1243. 10712703 10.1046/j.1365-2958.2000.01793.x

[27] Jung K, Krabusch M, Altendorf K (2001) Cs^+^ induces the *kdp* operon of *Escherichia coli* by lowering the intracellular K^+^ concentration. J Bacteriol 183: 3800–3803 10.1128/JB.183.12.3800-3803.2001 11371546 PMC95259

[28] Yan H, Fukamachi T, Saito H, Kobayashi H (2011) Expression and activity of Kdp under acidic conditions in *Escherichia coli* . Biol Pharm Bull 34: 426–429. 21372396 10.1248/bpb.34.426

[29] Jung K, Veen M, Altendorf K (2000) K^+^ and ionic strength directly influence the autophosphorylation activity of the putative turgor sensor KdpD of *Escherichia coli* . Journal of Biological Chemistry 275: 40142–40147 10.1074/jbc.M008917200 11016946

[30] Heermann R, Weber A, Mayer B, Ott M, Hauser E, et al (2009) The universal stress protein UspC scaffolds the KdpD/KdpE signaling cascade of *Escherichia coli* under salt stress. J Mol Biol 386: 134–148 10.1016/j.jmb.2008.12.007 19101563

[31] Lüttmann D, Heermann R, Zimmer B, Hillmann A, Rampp IS, et al (2009) Stimulation of the potassium sensor KdpD kinase activity by interaction with the phosphotransferase protein IIA^Ntr^ in *Escherichia coli* . Mol Microbiol 72: 978–994 10.1111/j.1365-2958.2009.06704.x 19400808

[32] Laermann V, Ćudić E, Kipschull K, Zimmann P, Altendorf K (2013) The sensor kinase KdpD of *Escherichia coli* senses external K^+^ . Mol Microbiol 88: 1194–1204 10.1111/mmi.12251 23651428

[33] Jung K, Altendorf K (1998) Individual substitutions of clustered arginine residues of the sensor kinase KdpD of *Escherichia coli* modulate the ratio of kinase to phosphatase activity. J Biol Chem 273: 26415–26420. 9756874 10.1074/jbc.273.41.26415

[34] Heermann R, Altendorf K, Jung K (2000) The hydrophilic N-terminal domain complements the membrane-anchored C-terminal domain of the sensor kinase KdpD of *Escherichia coli* . J Biol Chem 275: 17080–17085 10.1074/jbc.M000093200 10747873

[35] Heermann R, Lippert M-L, Jung K (2009) Domain swapping reveals that the N-terminal domain of the sensor kinase KdpD in *Escherichia coli* is important for signaling. BMC Microbiol 9: 133 10.1186/1471-2180-9-133 19589130 PMC2714519

[36] Kremling A, Heermann R, Centler F, Jung K, Gilles ED (2004) Analysis of two-component signal transduction by mathematical modeling using the KdpD/KdpE system of *Escherichia coli* . Biosystems 78: 23–37 10.1016/j.biosystems.2004.06.003 15555756

[37] Trchounian A, Kobayashi H (1999) Kup is the major K^+^ uptake system in *Escherichia coli* upon hyper-osmotic stress at a low pH. FEBS Letters 447: 144–148. 10214935 10.1016/s0014-5793(99)00288-4

[38] McLaggan D, Naprstek J, Buurman ET, Epstein W (1994) Interdependence of K^+^ and glutamate accumulation during osmotic adaptation of *Escherichia coli* . J Biol Chem 269: 1911–1917. 7904996

[39] Epstein W, Davies M (1970) Potassium-dependant mutants of *Escherichia coli* K-12. J Bacteriol 101: 836–843. 4908783 10.1128/jb.101.3.836-843.1970 PMC250399

[40] Heermann R, Zeppenfeld T, Jung K (2008) Simple generation of site-directed point mutations in the *Escherichia coli* chromosome using Red®/ET® Recombination. Microb Cell Fact 7: 14 10.1186/1475-2859-7-14 18435843 PMC2373285

[41] Bertrand J, Altendorf K, Bramkamp M (2004) Amino acid substitutions in putative selectivity filter regions III and IV in KdpA alter ion selectivity of the KdpFABC complex from *Escherichia coli* . J Bacteriol 186: 5519–5522 10.1128/JB.186.16.5519-5522.2004 15292155 PMC490938

[42] Nakamura T, Yuda R, Unemoto T, Bakker EP (1998) KtrAB, a new type of bacterial K^+^-uptake system from *Vibrio alginolyticus* . J Bacteriol 180: 3491–3494. 9642210 10.1128/jb.180.13.3491-3494.1998 PMC107312

[43] Rodriguez-Fernandez M, Banga JR (2010) SensSB: a software toolbox for the development and sensitivity analysis of systems biology models. Bioinformatics 26: 1675–1676 10.1093/bioinformatics/btq242 20444837

[44] Fritz G, Koller C, Burdack K, Tetsch L, Haneburger I, et al (2009) Induction kinetics of a conditional pH stress response system in *Escherichia coli* . J Mol Biol 393: 272–286 10.1016/j.jmb.2009.08.037 19703467

[45] Blattner FR, Plunkett G, Bloch CA, Perna NT, Burland V, et al (1997) The complete genome sequence of *Escherichia coli* K-12. Science 277: 1453–1462. 9278503 10.1126/science.277.5331.1453

[46] Epstein W, Kim BS (1971) Potassium transport loci in *Escherichia coli* K-12. J Bacteriol 108: 639–644. 4942756 10.1128/jb.108.2.639-644.1971 PMC247121

[47] Aiba H, Adhya S, de Crombrugghe B (1981) Evidence for two functional *gal* promoters in intact *Escherichia coli* cells. J Biol Chem 256: 11905–11910. 6271763

[48] Livak KJ, Schmittgen TD (2001) Analysis of relative gene expression data using real-time quantitative PCR and the 2(-Delta Delta C(T)) Method. Methods 25: 402–408 10.1006/meth.2001.1262 11846609

[49] Bossemeyer D, Borchard A, Dosch DC, Helmer GC, Epstein W, et al (1989) K^+^-transport protein TrkA of *Escherichia coli* is a peripheral membrane protein that requires other *trk* gene products for attachment to the cytoplasmic membrane. J Biol Chem 264: 16403–16410. 2674131

[50] Stock JB, Rauch B, Roseman S (1977) Periplasmic space in *Salmonella typhimurium* and *Escherichia coli* . J Biol Chem 252: 7850–7861. 334768

[51] Cayley S, Lewis BA, Guttman HJ, Record MT (1991) Characterization of the cytoplasm of *Escherichia coli* K-12 as a function of external osmolarity. Implications for protein-DNA interactions in vivo. J Mol Biol 222: 281–300. 1960728 10.1016/0022-2836(91)90212-o

[52] Fulton AB (1982) How crowded is the cytoplasm? Cell 30: 345–347. 6754085 10.1016/0092-8674(82)90231-8

[53] Laemmli UK (1970) Cleavage of structural proteins during the assembly of the head of bacteriophage T4. Nature 227: 680–685 10.1038/227680a0 5432063

[54] Lee SB, Bailey JE (1984) Genetically structured models forlac promoter-operator function in the *Escherichia coli* chromosome and in multicopy plasmids: Lac operator function. Biotechnol Bioeng 26: 1372–1382 10.1002/bit.260261115 18551662

[55] Nelder JA (1961) The fitting of a generalization of the logistic curve. Biometrics 17: 89–110.

[56] Rhoads DB, Epstein W (1978) Cation transport in *Escherichia coli*. IX. Regulation of K^+^ transport. J Gen Physiol 72: 283–295. 359759 10.1085/jgp.72.3.283 PMC2228538

