# Supplementary figures and images for: Dynamics of an Interactive Network Composed of a Bacterial Two-Component System, a Transporter and K+ as Mediator
Source: PLoS One. 2014 Feb 28;9(2):e89671. doi: 10.1371/journal.pone.0089671 (PMC3938482; doi:10.1371/journal.pone.0089671)

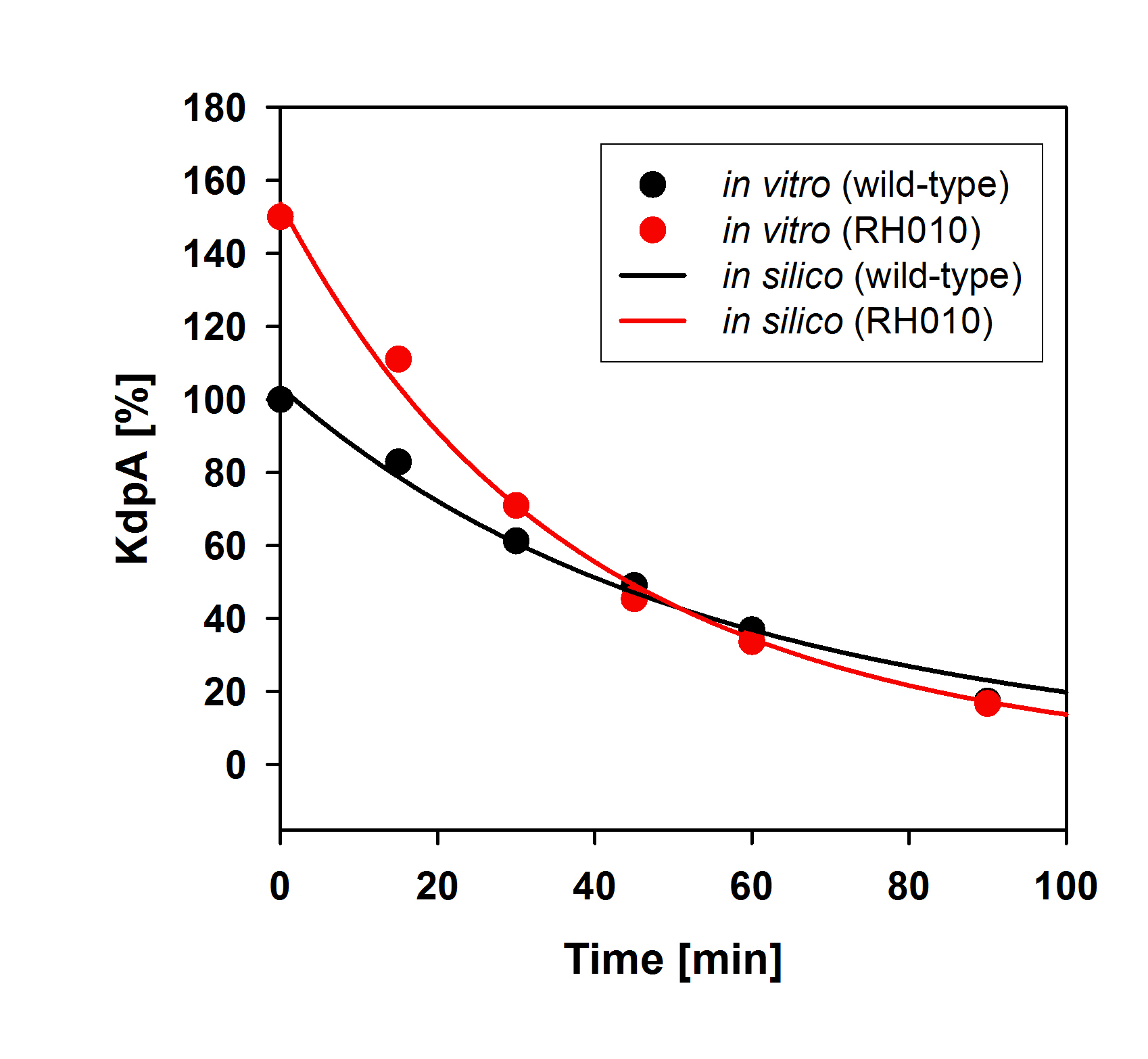

Supplement: Figure S1 — Proteolysis controls appropriate KdpFABC level. E. coli MG1655 rpsL150 (wild type) and E. coli RH010 were cultivated in phosphate buffered minimal medium containing 10 mM K+ up to the mid-logarithmic growth phase, exposed to extreme K+ limitation (0.04 mM K+) for 10 min to activate kdpFABC expression before proteins were labelled with 35S-methionine. Labelling of de novo synthesized proteins was quenched by adding an excess of non-labelled methionine after 10 min. Protein turnover is determined by the reduction of the labelled protein complex over the time. At different times, samples were taken, immunoprecipitated with αKdpFABC antiserum, subjected to SDS-PAGE, and the amount of KdpFABC was quantified from the autoradiographies of the gels. The data represent one of three independently performed characteristic experiments. Half-life was determined to be 36.7 min for wild type KdpFABC and 28.4 min KdpFA(G345S)BC. (TIF) [file pone.0089671.s001.tif]
