# Supplementary material for: Dynamics of an Interactive Network Composed of a Bacterial Two-Component System, a Transporter and K+ as Mediator
Source: PLoS One. 2014 Feb 28;9(2):e89671. doi: 10.1371/journal.pone.0089671 (PMC3938482; doi:10.1371/journal.pone.0089671)
Supplement: Table S1 — Total and viable cell numbers and cytoplasmic volume after exposure of E. coli to K+ limitation. E. coli MG1655 cells were grown in minimal medium containing 10 mM K+ to the mid-logarithmic growth phase and shifted to K+ limitation (40 µM K+). At the indicated times, cells were harvested, and the total and viable cell numbers as well as the cytoplasmic volume of the cells were determined as described in Materials and Methods. The values represent average values of at least three independent experiments. Standard deviations were below 5%. n.d., not determined. (DOCX) [file pone.0089671.s002.docx]

**Table S1.** Total and viable cell numbers and cytoplasmic volume after exposure of *E. coli* to K^+^ limitation. *E. coli* MG1655 cells were grown in minimal medium containing 10 mM K^+^ to the mid-logarithmic growth phase and shifted to K^+^ limitation (40 µM K^+^). At the indicated times, cells were harvested, and the total and viable cell numbers as well as the cytoplasmic volume of the cells were determined as described in Materials and Methods. The values represent average values of at least three independent experiments. Standard deviations were below 5 %. n.d., not determined.

| **Time [min]** | **Total number of cells exposed to**  **40 µM K^+^**  **[cells/ml]** | **Number of viable cells exposed to**  **40 µM K^+^**  **[cells/ml]** | **Cytoplasmic volume of cells exposed to 40 µM K^+^ [l/cell]** | **Cytoplasmic volume of cells exposed to 10 mM K^+^ [l/cell]**  **(control)** |
| --- | --- | --- | --- | --- |
| **1** | 4.60 x 10^8^ | 2.92 x 10^8^ | 1.12 x 10^-15^ | 8.99 x 10^-16^ |
| **5** | 5.68 x 10^8^ | 3.49 x 10^8^ | n.d. | n.d. |
| **10** | 5.12 x 10^8^ | 2.97 x 10^8^ | 8.07 x 10-16 | 8.36 x 10^-16^ |
| **15** | 5.12 x 10^8^ | 4.45 x 10^8^ | n.d. | n.d. |
| **20** | 6.25 x 10^8^ | 3.94 x 10^8^ | n.d. | n.d. |
| **25** | 5.38 x 10^8^ | 3.51 x 10^8^ | n.d. | n.d. |
| **30** | 5.76 x 10^8^ | 4.70 x 10^8^ | n.d. | 7.6 x 10^-16^ |
| **40** | 6.23 x 10^8^ | 5.10 x 10^8^ | n.d. | n.d. |
| **50** | 6.70 x 10^8^ | 5.62 x 10^8^ | n.d. | n.d. |
| **60** | 6.63 x 10^8^ | 5.57 x 10^8^ | 7.46 x 10^-16^ | 6.28 x 10^-16^ |
| **70** | 8.72 x 10^8^ | 7.02 x 10^8^ | n.d. | n.d. |
| **80** | 8.83 x 10^8^ | 7.21 x 10^8^ | n.d. | n.d. |
| **90** | 1.07 x 10^9^ | 8.09 x 10^8^ | n.d. | n.d. |
| **100** | 1.00 x 10^9^ | 7.80 x 10^8^ | n.d. | n.d. |
| **110** | 1.14 x 10^9^ | 1.00 x 10^9^ | n.d. | n.d. |
| **120** | 1.24 x 10^9^ | 1.04 x 10^9^ | 6.32 x 10^-16^ | 4.68 x 10^-16^ |
| **130** | 1.39 x 10^9^ | 1.11 x 10^9^ | n.d. | n.d. |
| **140** | 1.29 x 10^9^ | 9.58 x 10^8^ | n.d. | n.d. |
| **150** | 1.34 x 10^9^ | 9.21 x 10^8^ | n.d. | n.d. |
| **160** | 1.41 x 10^9^ | 9.06 x 10^8^ | n.d. | n.d. |
| **170** | 1.30 x 10^9^ | 1.02 x 10^9^ | n.d. | n.d. |
| **180** | 1.41 x 10^9^ | 9.46 x 10^8^ | 6.93 x 10^-16^ | 5.83 x 10^-16^ |
